# Editorial: Redox regulation in sperm and oocyte from gametogenesis to fertilization for reproductive health

**DOI:** 10.3389/fcell.2026.1775345

**Published:** 2026-01-14

**Authors:** Xue Du, Huaming Xi, Fa Ren

**Affiliations:** 1 Department of Reproductive Medicine, Affiliated Hospital of Shandong Second Medical University, Weifang, Shandong, China; 2 School of Clinical Medicine, Shandong Second Medical University, Weifang, Shandong, China; 3 College of Animal Science and Technology and College of Veterinary Medicine of Zhejiang A&F University, Hangzhou, China

**Keywords:** infertility, oocyte, oxidative stress, reproductive health, sperm

Human infertility, a growing public health issue, affects one in six couples worldwide. It is a multifactorial condition influenced by genetic, environmental, and lifestyle factors. (Sang et al., 2023). Consequently, gaining a comprehensive understanding of the molecular regulatory mechanisms governing gamete development and fertilization processes is crucial, particularly regarding the significant role of redox regulation.

This Research Topic, “*Redox regulation in sperm and oocyte from gametogenesis to fertilization for reproductive health*,” comprises five articles that examine the molecular mechanisms by which oxidation-reduction processes influence spermatogenesis. Additionally, it identifies novel biomarkers and explores treatment strategies pertinent to reproductive health. This Research Topic offers significant scientific insights for future research on gamete health and its effects on early embryonic development.

Oxidative stress (OS) refers to an imbalance between the body’s oxidative and antioxidant systems under normal physiological conditions. The excessive production of reactive oxygen species (ROS) during OS can result in oxidative damage, which may lead to apoptosis, tissue injury, and various diseases (Kelley et al., 2025). However, due to the complexity of the underlying mechanisms of OS, further exploration is necessary to investigate its role and treatment strategies within the realm of reproductive health.

During spermatogenesis, the microenvironment within the testes is intricately regulated by redox states. There were several novel markers evaluated in this Research Topic. Feng et al. systematically elucidated the central hub role of Sertoli cells in testicular development and male reproduction. Sertoli cells are not only a structural and nutritional support cell for spermatogenesis but also regulate the differentiation and function of Leydig cells, peritubular muscle-like cells (PMCs), and testicular macrophages (TMs) by secreting various factors, helping to maintain the stability of the testicular microenvironment. The relationship between Sertoli cell dysfunction and male infertility, particularly in conditions such as Sertoli-only syndrome, as well as the effects of endocrine disruptors and aging, underscores the urgent need for further in-depth research into their regulatory mechanisms. Employing innovative technologies in future studies will be essential to elucidate these complex interactions (Tahmasbpour et al., 2022). Liu et al. investigated how L-cysteine alleviates testicular injury induced by busulfan in mice by regulating the CBS/H2S axis and PI3K/Akt/mTOR pathway. Their findings demonstrate that L-cysteine can enhance sperm count, motility, testicular weight, and structural integrity while also mitigating oxidative stress and cell apoptosis and repairing the damaged blood-testis barrier. These results suggest that L-cysteine may serve as a promising therapeutic strategy for the prevention and treatment of chemotherapy-related male infertility. Additionally, Liu et al. examined the effects of polystyrene nanoplastics on the reproductive function of male mice. Research has shown that oral exposure to polystyrene nanoplastics can trigger immune inflammatory responses in testicular tissue and reduce sperm motility and metabolic function, particularly affecting glycerophospholipid biosynthesis and DNA repair pathways. In addition, this exposure is associated with abnormal early embryonic development, disrupted gene expression, and increased oxidative stress and DNA damage in embryos, revealing the transgenerational effects of nanoplastics on embryonic development mediated by sperm. In summary, maintaining the redox balance during spermatogenesis is key to ensuring male fertility.

Similarly, OS plays a critical role in multiple aspects of female reproduction, including ovulation, endometrium decidualization, menstruation, oocyte fertilization, and embryonic development and implantation (Vaskova et al., 2023). ROS regulate numerous physiological functions within the reproductive tract; however, excessive levels can lead to significant pathologies that adversely affect female reproductive health. The redox status can influence early embryo development by modifying key transcription factors and gene expression. ROS concentrations may also play a major role in both oocyte fertilization and implantation. Jiang et al. systematically reviewed the protective effects of coenzyme Q10 (CoQ10) on female fertility and its application in assisted reproductive technology (ART). CoQ10, through its powerful antioxidant properties and key regulation of mitochondrial energy metabolism, can effectively improve oocyte quality, delay ovarian aging, and promote embryonic development, thereby providing theoretical basis and guidance for the clinical application of CoQ10 in reproductive medicine (Shang et al., 2024).

In summary, redox regulation is integral to the entire process of gamete development, maturation, fertilization, and early embryonic development, serving as the fundamental biological axis that sustains reproductive health. From mechanistic research to clinical translation, a systematic analysis of redox balance not only addresses infertility issues but also provides scientific support for improving population fertility quality.
